# Cardiometabolic and renal phenotypes and transitions in the United States population

**DOI:** 10.1038/s44161-023-00391-y

**Published:** 2023-12-15

**Authors:** Victor P. F. Lhoste, Bin Zhou, Anu Mishra, James E. Bennett, Sarah Filippi, Perviz Asaria, Edward W. Gregg, Goodarz Danaei, Majid Ezzati

**Affiliations:** 1 Department of Epidemiology and Biostatistics, School of Public Health, Imperial College London, London, UK; 2 MRC Centre for Environment and Health, School of Public Health, Imperial College London, London, UK; 3 Abdul Latif Jameel Institute for Disease and Emergency Analytics, Imperial College London, London, UK; 4 Department of Mathematics, Imperial College London, London, UK; 5 School of Population Health, Royal College of Surgeons in Ireland, Dublin, Ireland; 6 Department of Global Health and Population, Harvard T.H. Chan School of Public Health, Boston, MA, USA; 7 Department of Epidemiology, Harvard T.H. Chan School of Public Health, Boston, MA, USA; 8 Regional Institute for Population Studies, University of Ghana, Accra, Ghana

## Abstract

Cardiovascular and renal conditions have both shared and distinct determinants. In this study, we applied unsupervised clustering to multiple rounds of the National Health and Nutrition Examination Survey from 1988 to 2018, and identified 10 cardiometabolic and renal phenotypes. These included a ‘low risk’ phenotype; two groups with average risk factor levels but different heights; one group with low body-mass index and high levels of high-density lipoprotein cholesterol; five phenotypes with high levels of one or two related risk factors (‘high heart rate’, ‘high cholesterol’, ‘high blood pressure’, ‘severe obesity’ and ‘severe hyperglycemia’); and one phenotype with low diastolic blood pressure (DBP) and low estimated glomerular filtration rate (eGFR). Prevalence of the ‘high blood pressure’ and ‘high cholesterol’ phenotypes decreased over time, contrasted by a rise in the ‘severe obesity’ and ‘low DBP, low eGFR’ phenotypes. The cardiometabolic and renal traits of the US population have shifted from phenotypes with high blood pressure and cholesterol toward poor kidney function, hyperglycemia and severe obesity.

Diabetes, dementia, cardiovascular disease (CVD) and chronic kidney disease (CKD) are leading causes of death in the United States, in other high-income nations and, increasingly, in low-income and middle-income countries^1,2^. Obesity, short stature, high blood pressure, high heart rate, hyperglycemia, non-optimal lipid profiles and poor kidney function are established risk factors for one or more of these diseases^3–15^ and, in some cases, for infections such as coronavirus disease 2019 (ref. 16). As a result, people who have optimal levels of all or most risk factors are at low risk of cardiovascular and renal disease and cancer and vice versa^17–21^. Physiological risk factors can have complex correlations and co-occurrence patterns for at least two reasons. First, these physiological factors have shared as well as distinct genetic, behavioral, environmental and dietary determinants. For example, consumption of fruits and vegetables, meat, dairy, unsaturated versus saturated fats, processed versus whole grain carbohydrates and alcohol affect multiple cardiometabolic and renal traits beneficially or adversely, whereas others, such as sodium and potassium, affect only one or two traits (blood pressure and kidney function)^22–29^. Furthermore, these factors may cluster differently among different subgroups of a population^30^ and change over time^31^. Second, some of these physiological risk factors are themselves etiologically related; for example, obesity is a risk factor for dyslipidemia, elevated blood pressure and hyperglycemia^32,33^.

At the population level, some studies have quantified trends in individual cardiometabolic risk factors in the US population, other countries or globally^34–43^. Other studies have counted the number of cardiometabolic risk factors^44,45^, with some also quantifying association with the risk of coronary heart disease^45^. Some studies have used concepts such as metabolic syndrome^46^, optimal cardiometabolic health^44^ and metabolically healthy obesity^47–49^ to identify groups of people with a specific pre-determined risk factor profile. Studies that used data-driven methods to identify cardiometabolic phenotypes were mostly based on data from specific subgroups of a population (for example, older adults)^50^, users of specific health programs^51^ or people with a specific index disease, such as diabetes^52–54^, sepsis^55^ or cardiogenic shock^56^. The only study analyzing health-related phenotypes in an entire national population^57^ used a mix of behavioral, physiological and diagnostic variables at a single point in time for methodological assessment; it did not analyze change over time or the clinical or epidemiological characteristics of the clusters. Beyond cardiometabolic and renal health, some studies identified co-occurrences, or subtypes, of specific diseases in large cohorts, such as the UK Biobank^58^, in primary care patients from different countries^59,60^, especially using electronic health records^61–67^. These studies used a range of clustering methods^66,68^.

In the present study, we applied a data-driven approach to repeated nationally representative health examination surveys, namely the National Health and Nutrition Examination Survey (NHANES), from 1988 to 2018, to identify a comprehensive set of cardiometabolic and renal phenotypes in the United States adult population. We measured how the prevalence of these phenotypes has changed over time and characterized their sociodemographic, epidemiological and clinical predictors. This information is needed for planning and priority setting for population-based prevention programs and health system interventions to coherently and effectively prevent and manage conditions based on their co-occurrence in the population^69,70^.

## Cardiometabolic and renal phenotypes of the US population

**Fig. 1:**
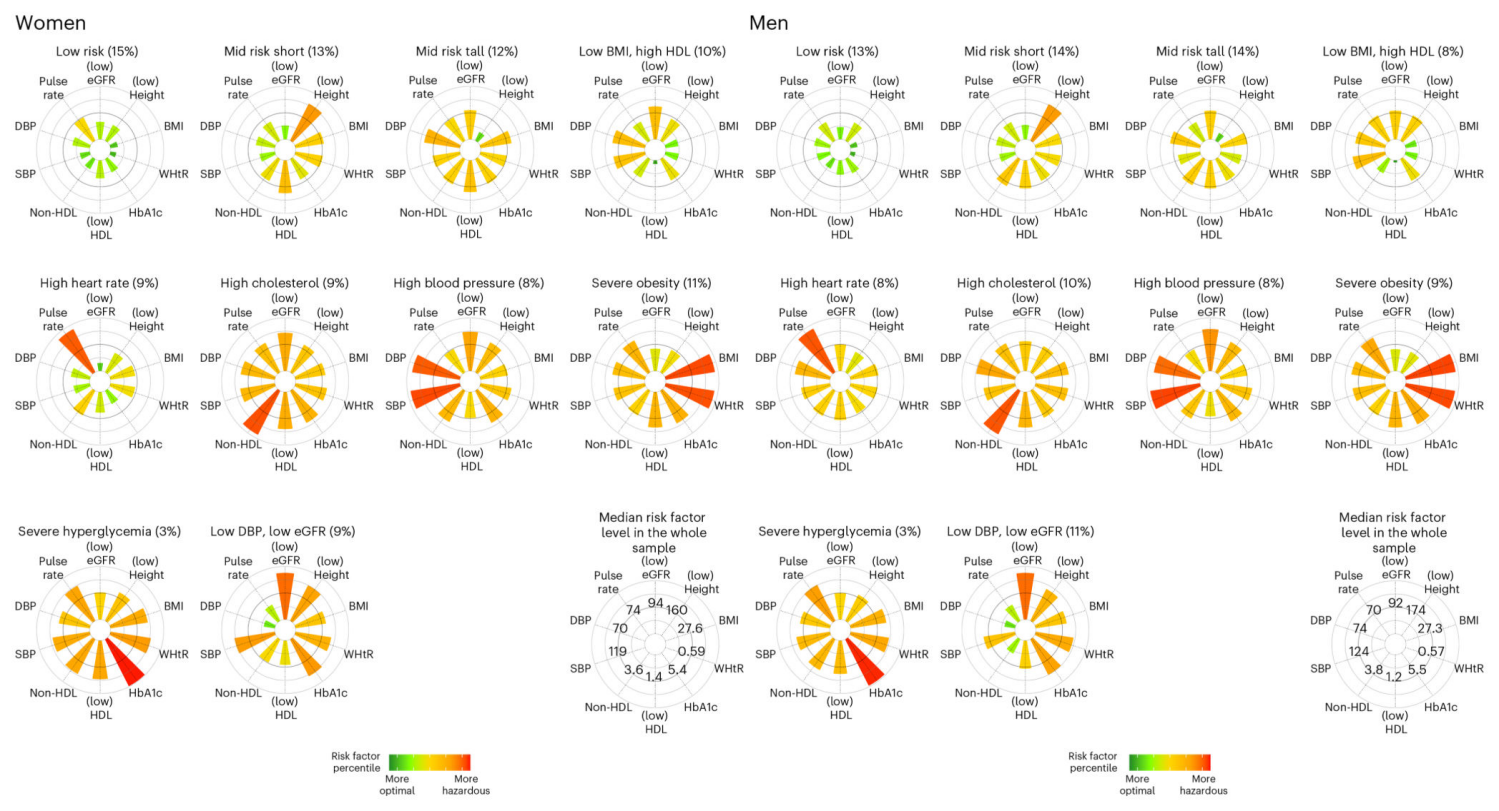
Risk factor profiles of the cardiometabolic and renal clusters of US adults for women and men. Each panel corresponds to a cluster; each bar shows the median value of one risk factor for all participants in the cluster. The number next to the cluster name represents the percentage of the participants grouped in this cluster. The concentric circles show the minimum, 25th, 50th and 75th percentiles and maximum in the whole sample, with the median shown in darker color. The height and color of the bar represent the median level of each risk factor, positioned relative to the distribution in the whole population, so that the scale is common across all clusters. The bottom-right panel shows the median value for each risk factor in the whole sample, and Supplementary Table 1 shows other percentiles for each risk factor in each cluster. The scale is reversed for height, eGFR and HDL because lower values indicate higher risk.

We identified 10 clusters (phenotypes) for both men and women that collectively characterized the cardiometabolic and renal traits of the US population from 1988 to 2018 (Fig. 1). The reasons for using 10 clusters are stated in the Methods, and the results with other cluster numbers are presented below. The identified phenotypes were similar between men and women, even though we analyzed data for the two sexes separately.

For both sexes, we identified a ‘low risk’ phenotype with near-optimal risk factor levels, accounting for 15% and 13% of the sample for women and men, respectively. We also identified two clusters (‘mid risk short’ and ‘mid risk tall’) jointly accounting for 25% and 28% of the sample for women and men, respectively, with risk factor levels mostly around sample medians. These two clusters differed by their average height and, to a lesser extent, by blood pressure and estimated glomerular filtration rate (eGFR) levels, with the ‘mid risk short’ cluster having, on average, shorter height (median of 155 cm versus 167 cm for women; 168 cm versus 182 cm for men) (Supplementary Table 1), lower blood pressure and higher eGFR than the ‘mid risk tall’ cluster. We also identified a group (‘low BMI, high HDL’) characterized by low levels of body mass index (BMI) and waist-to-height ratio (WHtR) and high high-density lipoprotein (HDL) cholesterol relative to the rest of the NHANES sample but with other risk factors being around the sample median.

**Extended Data Fig. 1:**
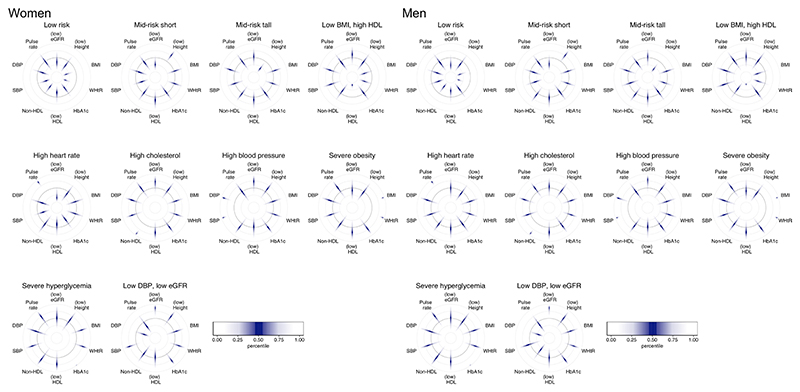
Risk factor distribution within each cluster. Each panel corresponds to a cluster, the color shows the distribution of each variable in each cluster with darker color at the center of the distribution. The concentric circles show the minimum, 25^th^, 50^th^, 75^th^ percentiles and maximum in the whole sample, with the median shown in darker color. Each percentile is positioned relative to the distribution in the whole population so that the scale is common across all clusters. The scale is reversed for height, eGFR and HDL because lower values indicate higher risk. eGFR: estimated glomerular filtration rate; BMI: body-mass index; WHtR: waist-to-height ratio; HbA1c: glycated hemoglobin; HDL: high-density lipoprotein cholesterol; non-HDL: non-high-density lipoprotein cholesterol; SBP: systolic blood pressure; DBP: diastolic blood pressure.

Five clusters were characterized by having high levels of one or two related risk factors accounting together for 40% of the sample for both sexes. These were ‘high cholesterol’, ‘high blood pressure’, ‘severe hyperglycemia’, ‘high heart rate’ and ‘severe obesity’. For instance, the ‘severe hyperglycemia’ phenotype had a median glycated hemoglobin (HbA1c) of 9.9% for women and 9.8% for men, but their median BMI (and WHtR) was much lower than those of the ‘severe obesity’ cluster (median BMI of 31.8 kg m^−2^ and 29.7 kg m^−2^ in the ‘severe hyperglycemia’ cluster for women and men, respectively, compared to a median BMI of 41.1 kg m^−2^ and 38.2 kg m^−2^ in the ‘severe obesity’ cluster). Similarly, the ‘high blood pressure’ cluster had a median systolic blood pressure (SBP) of 159 mmHg for both sexes, and the ‘high cholesterol’ cluster had a median non-HDL cholesterol of 5.5 mmol L^−1^ for both women and men, with other risk factor levels lying between the median and 75th percentiles of the entire NHANES sample. In all these clusters, the defining risk factor varied less among member participants than the other risk factors (Extended Data Fig. 1), further illustrating that its high value was the shared feature among participants who fell in the cluster. Finally, in both sexes, the last cluster (‘low DBP, low eGFR’) was characterized by low levels of diastolic blood pressure (DBP) and eGFR. For example, women who fell in the ‘low DBP, low eGFR’ cluster had a median DBP of 61 mmHg and a median eGFR of 63 ml/min/1.73 m^2^.

## Demographic and clinical characteristics of clusters

**Extended Data Fig. 2:**
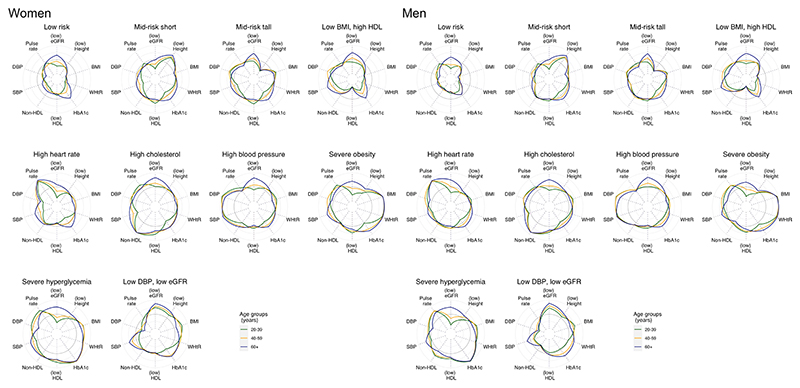
| Risk factor levels by age group in cardiometabolic and renal clusters. Each panel corresponds to a cluster, each line shows the median value of one biomarker for one age group within each cluster. The concentric circles show the minimum, 25^th^, 50^th^, 75^th^ percentiles and maximum in the whole sample, with the median shown in darker color. Each line is positioned relative to the distribution in the whole population so that the scale is common across all clusters and age groups. The scale is reversed for height, eGFR and HDL because lower values indicate higher risk. eGFR: estimated glomerular filtration rate; BMI: body-mass index; WHtR: waist-to-height ratio; HbA1c: glycated hemoglobin; HDL: high-density lipoprotein cholesterol; non-HDL: non-high-density lipoprotein cholesterol; SBP: systolic blood pressure; DBP: diastolic blood pressure.

Most of the identified cardiometabolic and renal phenotypes had a mix of young (20–39 years), middle-aged (40–59 years) and old (60 years and older) adults. The exceptions were two clusters for men and three for women with predominantly young people (‘low risk’ and ‘mid risk short’ for both sexes and ‘high heart rate’ for women) and one with predominantly old people (‘low DBP, low eGFR’) (Table 1). Even though 73% of women and 77% of men in the ‘low risk’ phenotype were aged 20–39 years, 4% and 6%, respectively, were older than 60 years with near-optimal risk factor profiles similar to their younger peers, except for slightly lower eGFR and higher HbA1c. Similarly, although most (92% of women and 90% of men) in the cluster ‘low DBP, low eGFR’ were 60 years or older, a small percentage (1% and 2%, respectively) were aged 20–39 years. Within each cluster, individuals of different age groups generally had similar risk factor profiles, especially on the defining risk factors in the higher risk phenotypes (Extended Data Fig. 2).

The ‘low risk’ group had the lowest number of morbidities and medication use (Table 1 and Extended Data Table 1). As expected, 96% of women and 98% of men in the ‘high blood pressure’ cluster had hypertension, yet this condition was also prevalent in ≥50% of participants in some other clusters—for example, ‘low DBP, low eGFR’ and ‘severe hyperglycemia’ for both sexes and ‘severe obesity’ phenotype for men (most of those with hypertension in the ‘low DBP, low eGFR’ cluster had isolated systolic hypertension). Similarly, all participants in the ‘severe hyperglycemia’ cluster had diabetes; the next highest diabetes prevalence was in the ‘low DBP, low eGFR’ cluster (31% in both sexes), with the ‘severe obesity’ cluster having only the third highest prevalence (22% in women and 25% in men). Median HbA1c of people with diabetes in the ‘severe obesity’ cluster (6.88% for men and 6.77% for women) was much lower than median HbA1c of those in the ‘severe hyperglycemia’ cluster (9.9% for women and 9.8% for men). Finally, those in the ‘low DBP, low eGFR’ phenotype more frequently had a history of myocardial infarction (MI), stroke and congestive heart failure (CHF) than the other phenotypes—for example, 19% of men in this phenotype had a history of MI compared to 6% in the whole sample; similarly, 12% of men in this phenotype had a previous history of CHF compared to 4% in the whole sample.

The use of statins was relatively low in the ‘high cholesterol’ group—13% for women and 8% for men—with that of men being lower than the overall NHANES sample (Table 1). In contrast, statin and antihypertensive use was high in the ‘low DBP, low eGFR’ and ‘severe hyperglycemia’ groups (26–41% of participants in different cluster–sex combinations, which is 2–3 times more than in the overall samples), consistent with the clinical guidelines that recommend the use of these medicines among people with diabetes and history of MI and stroke, especially in older ages. In the ‘severe obesity’ cluster, antihypertensive and statin use was above average, which may partly account for this group having blood pressure and cholesterol levels around the population median. The use of most medicines was higher in the 2011–2018 period than over the entire analysis period, with the largest increase being that of statins (Extended Data Table 2). The increase in statin use was, however, less pronounced in the ‘high cholesterol’ phenotype (+38% relative increase for women and +4% for men) than in the whole sample (+48% for women and +45% for men), demonstrating that this phenotype was characterized by insufficiently treated or controlled levels of non-HDL cholesterol.

## Trends over time

**Extended Data Fig. 3:**
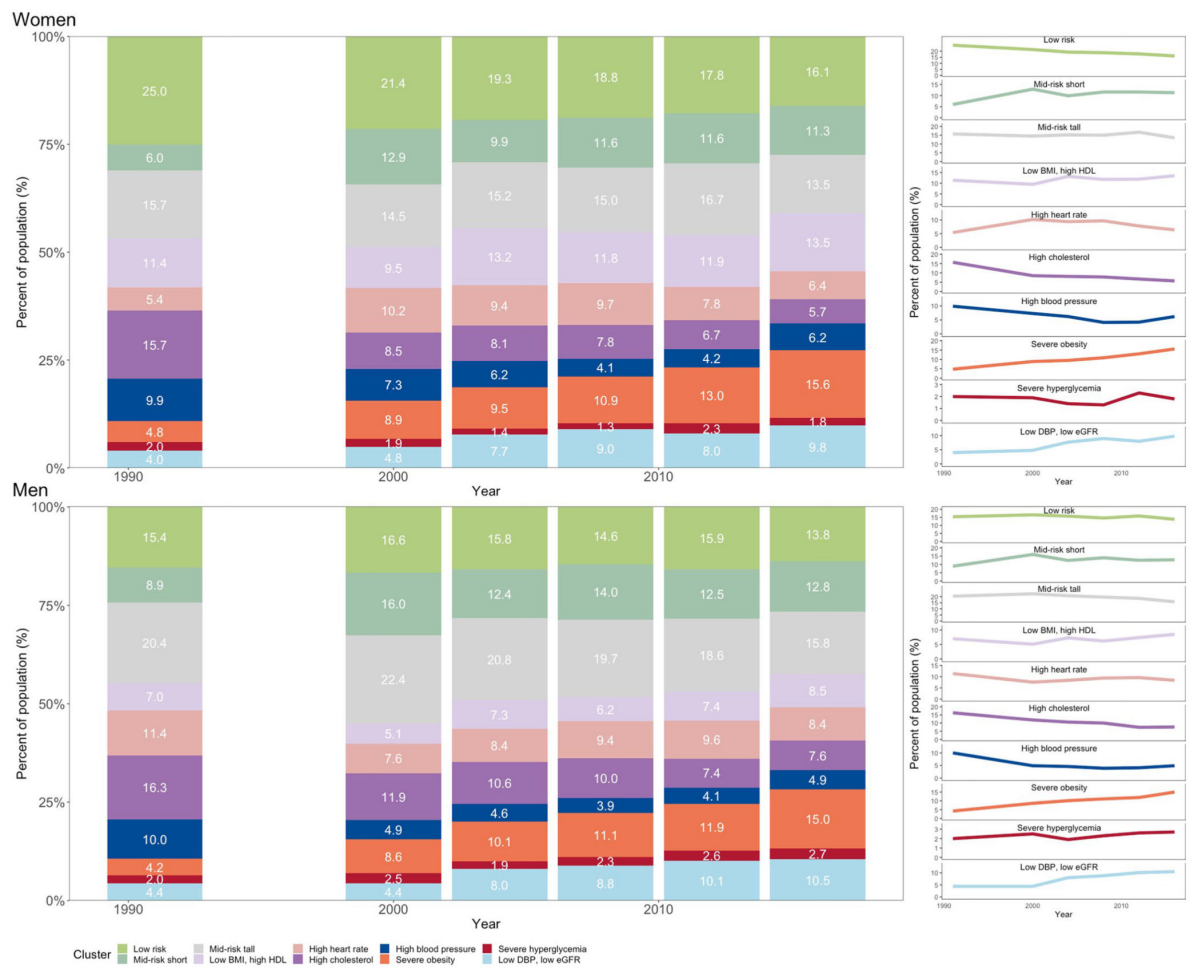
Trends in crude prevalence of cardiometabolic and renal clusters from 1988 to 2018. Crude prevalence was calculated as overall prevalence in each NHANES round without any adjustment for the age structure of the participants.

**Fig. 2:**
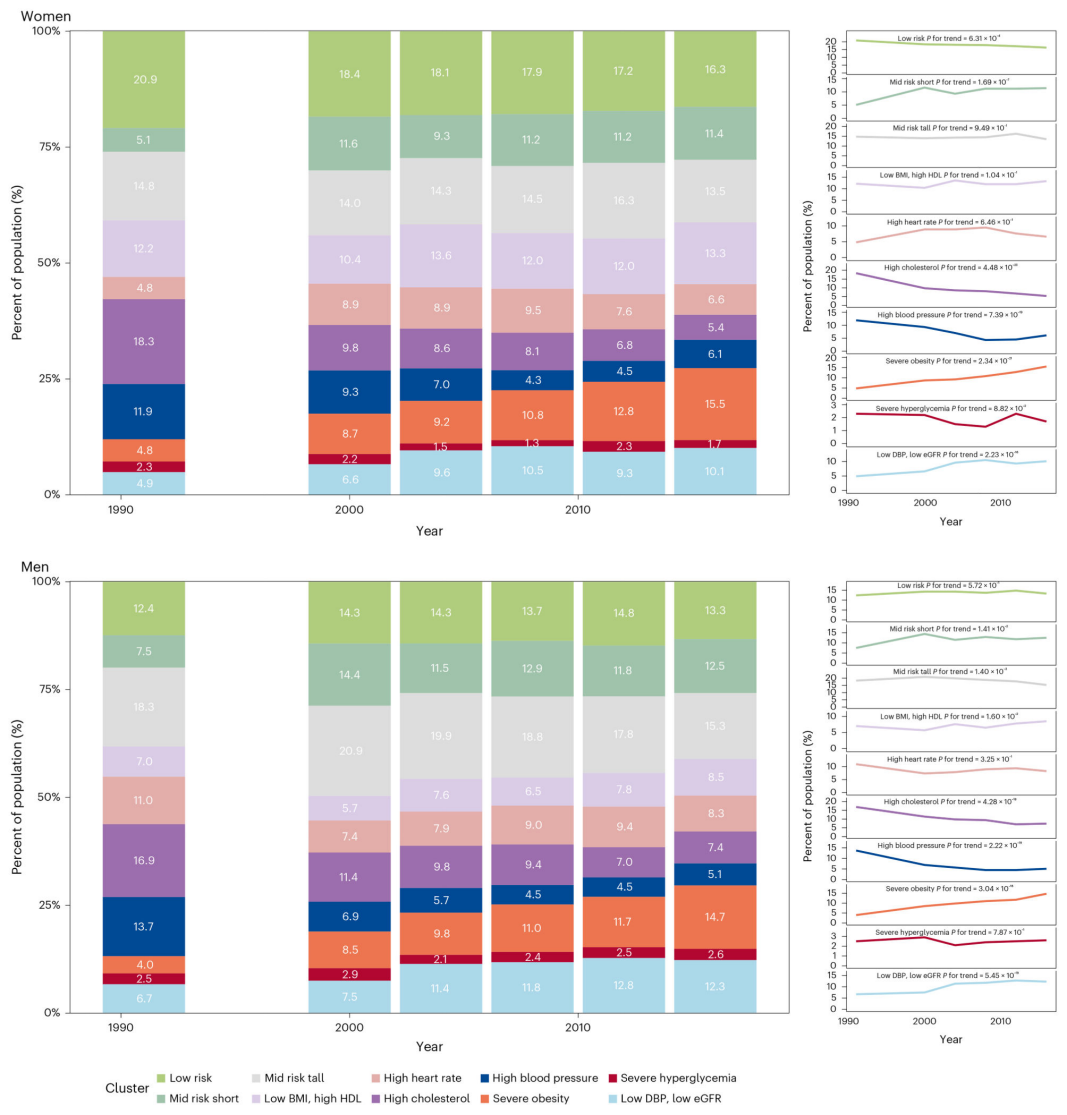
Trends in cardiometabolic and renal clusters from 1988 to 2018. The *P* values for trends were obtained from two-sided *t*-test from a logistic regression using the cluster assignment of individual participants, with adjustment for age as described in the Methods. No adjustments were made for multiple comparisons. The figure shows age-standardized prevalence for all clusters (bar charts) as well as individual clusters (lines). See Extended Data Fig. 3 for trends in crude prevalence. See Extended Data Table 3 for trends in pre-specified periods.

The cardiometabolic and renal risk profile of the US population changed from 1988 to 2018 (Fig. 2). The age-standardized prevalence of the ‘severe obesity’ phenotype more than tripled for both sexes and that of the ‘low DBP, low eGFR’ phenotype almost doubled over the entire analysis period. Most of the increase of the ‘low DBP, low eGFR’ phenotype occurred between 2000 and 2010, before plateauing after 2010 (*P* value for trend from 2010 to 2018 was 0.96 for women and 0.97 for men; Extended Data Table 3). In contrast, the prevalence of the ‘high blood pressure’ and ‘high cholesterol’ phenotypes more than halved in both sexes (*P* value for trend was <0.0001 for both sexes over the entire analysis period). However, since the late 2000s, there has been a reversal of the earlier declines in the prevalence of the ‘high blood pressure’ phenotype (*P* value for increasing trend from 2010 to 2018 was 0.0015 for women and 0.0346 for men). There was no statistically detectable change in the ‘severe hyperglycemia’ phenotype (*P* = 0.09 for women and 0.79 for men), which indicates that, despite the increase in the prevalence of diabetes in the United States, those at extreme values of HbA1c were stable. Rather, many of the additional people with diabetes fell in the ‘severe obesity’ and ‘low DBP, low eGFR’ clusters for which the prevalence increased over time. Most trends were consistent between the two sexes. A notable exception was the ‘low risk’ phenotype, which remained constant for men but decreased by 4.5 percentage points for women (*P* value for trend was 0.0006 over the entire analysis period), even though its prevalence remained higher in women than men throughout the analysis period. Trends in crude prevalence were nearly identical to the age-standardized trends (Extended Data Fig. 3).

## Changes in age patterns of clusters

**Fig. 3:**
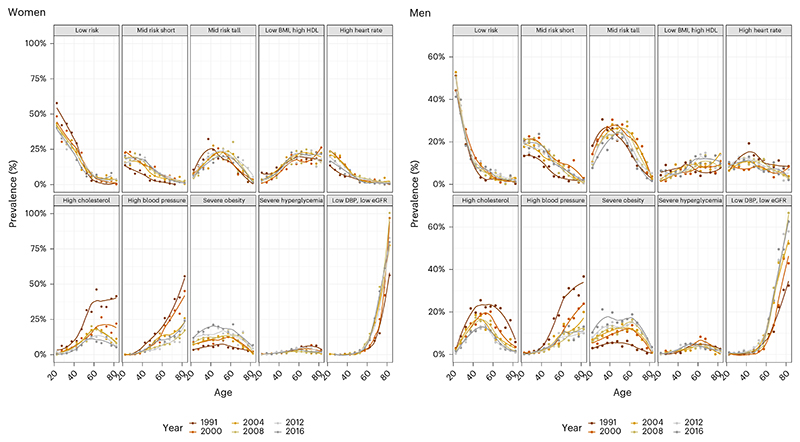
Age patterns of cardiometabolic and renal clusters. Each point represents the prevalence of a cluster for an age group from a survey mid-year. The color of the point represents the year of the survey. The lines represent the fitted local polynomial regression for each survey round.

The various cardiometabolic and renal phenotypes had differing age associations (Fig. 3). The ‘low risk’ and ‘mid risk short’ phenotypes for both sexes, and the ‘high heart rate’ phenotype for women, were more common among younger adults, and their prevalence decreased with age, with a much steeper age association for the ‘low risk’ group. Conversely, the ‘low DBP, low eGFR’ and ‘high blood pressure’ phenotypes became more prevalent throughout the life course, with a steeper age association for the ‘low DBP, low eGFR’ group. Other phenotypes tended to peak in middle ages.

Both ‘high blood pressure’ and ‘high cholesterol’ phenotypes decreased sharply in people aged 50 years and older from 1991 to 2008, likely due to the increased use of statins and antihypertensive medication; however, the decreases may have slowed down or stagnated in the past decade. In contrast, for both sexes, the age association of the ‘low DBP, low eGFR’ phenotype became steeper over time.

## Predictors of cardiometabolic and renal traits

**Fig. 4:**
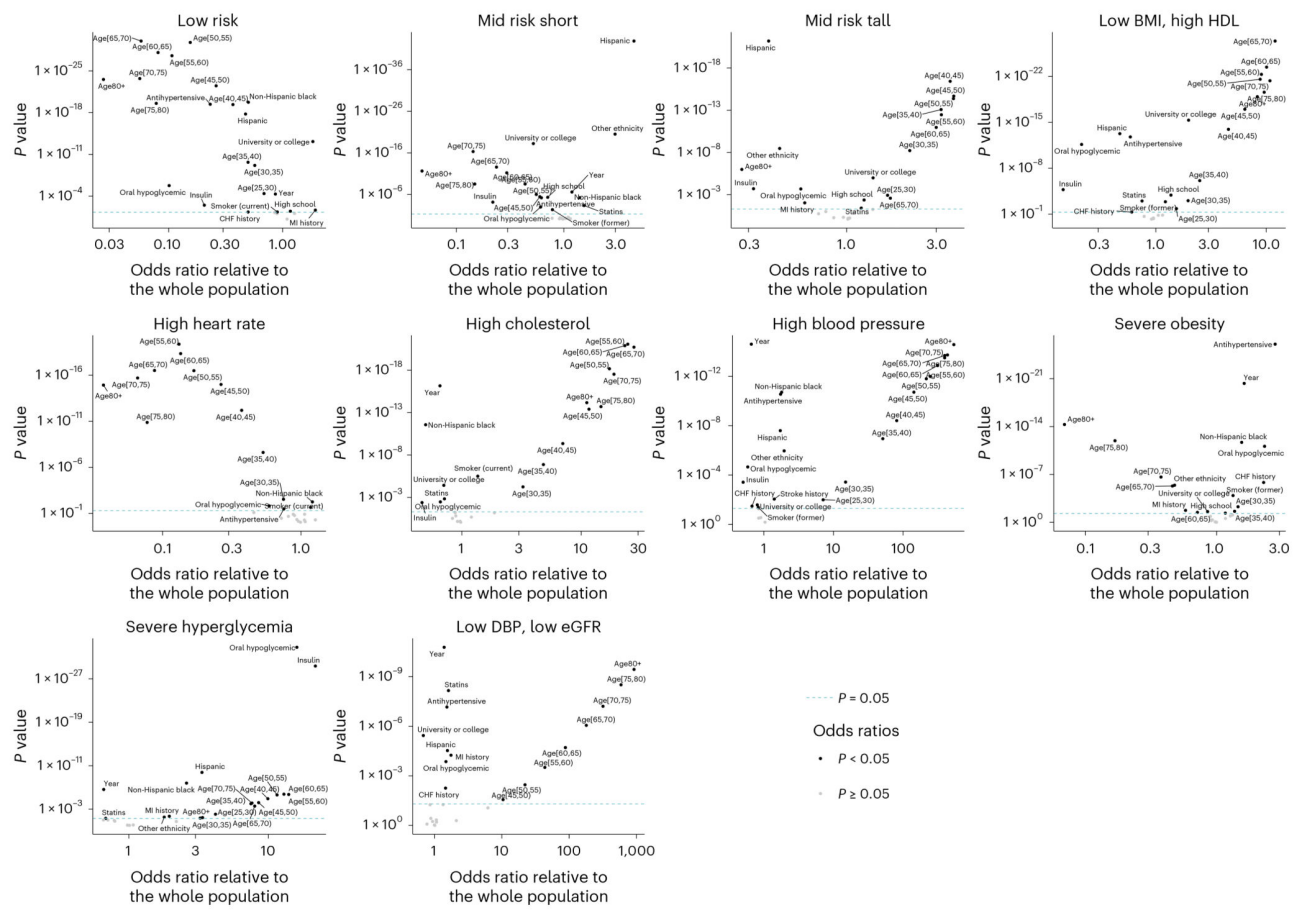
Predictors of the allocation to cardiometabolic and renal phenotypes in women. Each point shows one predictor used in the multivariable logistic regressions, as described in the Methods, with its position indicating its coefficient and *P* value obtained from a two-sided *t*-test and not adjusted for multiple comparison. Predictors with *P* < 0.05 are labeled. The reference categories were: 20–25-year-old individuals for age group, non-Hispanic White for ethnicity, below high school for education and never-smokers for smoking. The year coefficient represents changes in odds per decade.

**Fig. 5:**
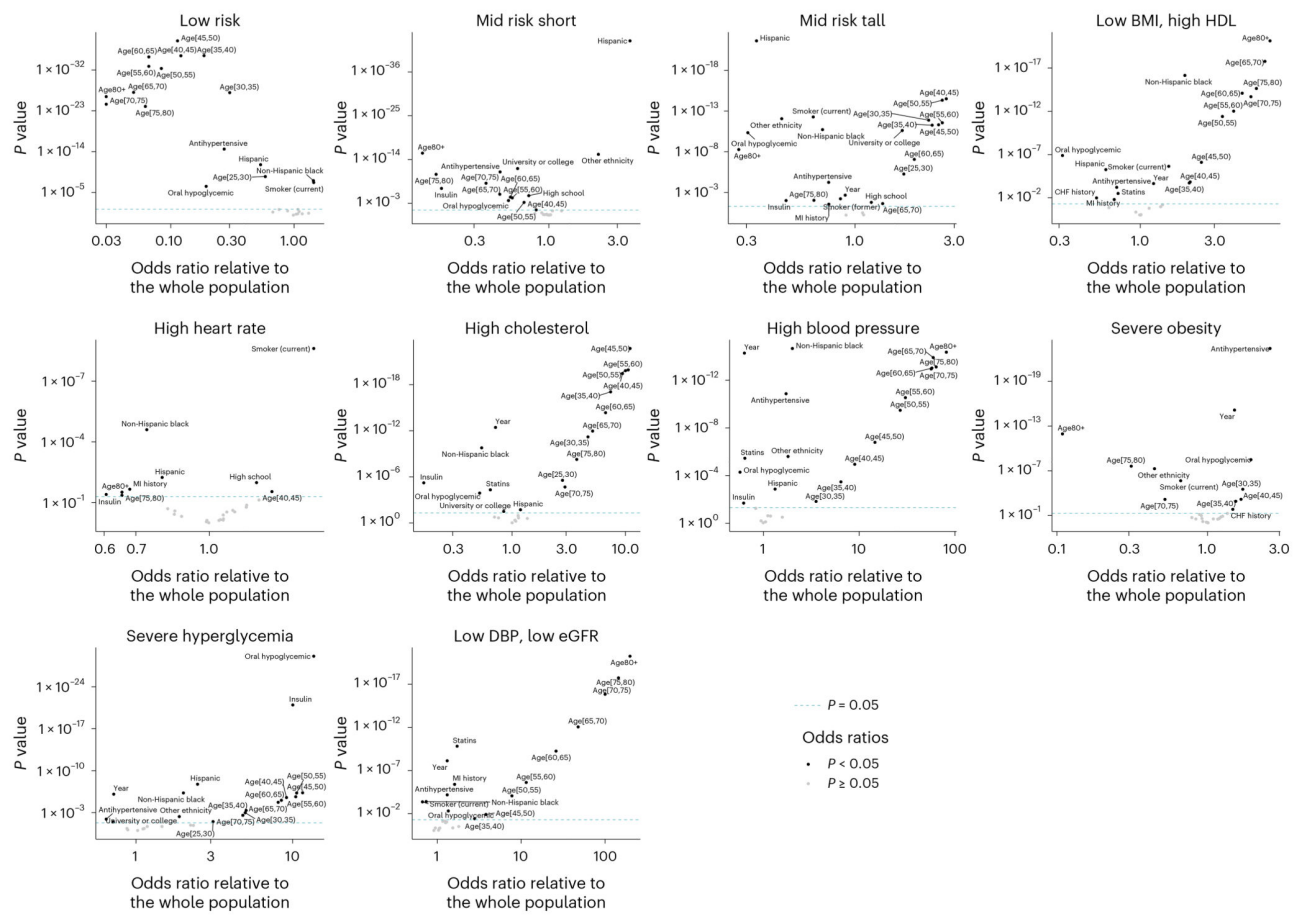
Predictors of the allocation to cardiometabolic and renal phenotypes in men. Each point shows one predictor used in the multivariable logistic regressions, as described in the Methods, with its position indicating its coefficient and *P* value obtained from a two-sided *t*-test and not adjusted for multiple comparison. Predictors with *P* < 0.05 are labeled. The reference categories were: 20–25-year-old individuals for age group, non-Hispanic White for ethnicity, below high school for education and never-smokers for smoking. The year coefficient represents changes in odds per decade.

We analyzed the sociodemographic, behavioral and clinical predictors of cluster membership in multivariate regressions as described in the Methods. Both education and ethnicity were associated with the partition of the participants into some of the cardiometabolic and renal phenotypes. Higher education was associated with lower odds of allocation to the ‘high cholesterol’ phenotype for both men and women, lower odds of allocation to the ‘severe hyperglycemia’ phenotype for men and lower odds of allocation to the ‘low DBP, low eGFR’ phenotype for women; it was associated with higher odds of being in the ‘low risk’ phenotype for women (Figs. 4 and 5). Hispanic and non-Hispanic Black women and men had higher odds of belonging to the ‘severe hyper-glycemia’ and ‘high blood pressure’ phenotypes than non-Hispanic Whites; Hispanic and non-Hispanic Black women had lower odds of belonging to the ‘low risk’ phenotype than non-Hispanic Whites; and non-Hispanic Black men and women had lower odds of belonging to the ‘high cholesterol’ phenotype.

The use of statins was associated with lower odds of belonging to the ‘high cholesterol’ phenotype for both men and women, demonstrating its effectiveness in controlling hypercholesterolemia. In contrast, diabetes medications, both oral and insulin, were associated with the ‘severe hyperglycemia’ phenotype in both sexes, as were antihypertensive medications for the ‘high blood pressure’ phenotype, albeit with a smaller magnitude than the former association. This shows that many individuals in these two phenotypes have uncontrolled diabetes or hypertension despite being treated^41^. Individuals on antihypertensive medicines also had higher odds of belonging to the ‘severe obesity’ phenotype, which provides one explanation for this group having a blood pressure level around the population median, despite the association between obesity and hypertension^33^. We also found that previous history of MI (both sexes) as well as previous history of CHF (women) were associated with the ‘low DBP, low eGFR’ phenotype even after adjusting for age and other predictors.

## Influence of the number of clusters

**Fig. 6:**
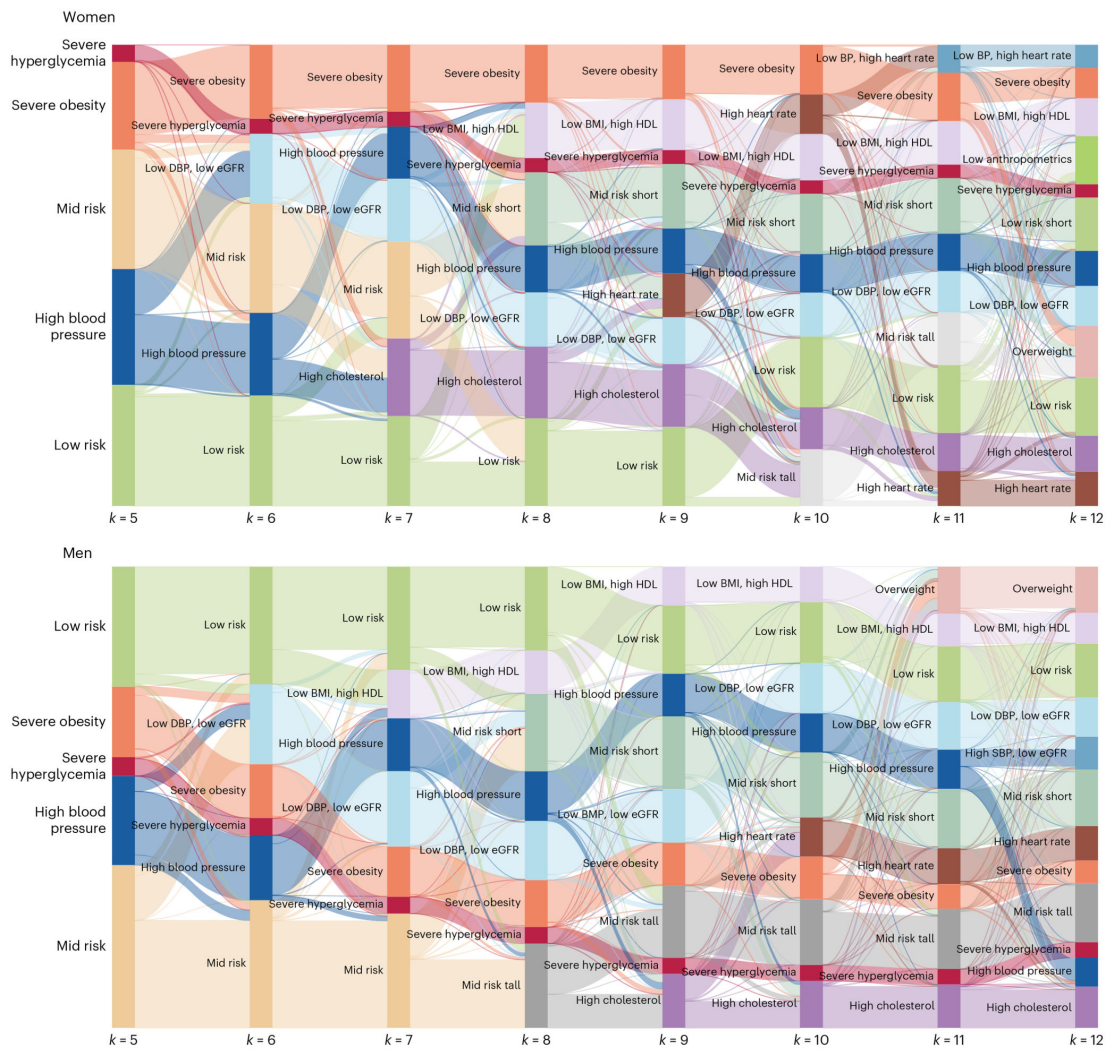
Changes in cardiometabolic and renal clusters in relation to the number of clusters. Each segment corresponds to a phenotype identified for a specific number of clusters (*k*), as *k* changes from 5 to 12. For each *k*, the vertical height shows the cluster prevalence, and the clusters were named based on their risk factor levels, as seen in Supplementary Fig. 1. The flow between segments indicates how clusters partition and merge as the number of clusters changes.

As described in the Methods, while our main results are based on 10 clusters we also investigated cluster membership and characteristics when sequentially changing the number of clusters (*k*) from 5 to 12. Even with five clusters (*k* = 5), four epidemiologically relevant cardio-metabolic and renal phenotypes were identified—‘low risk’, ‘severe hyperglycemia’, ‘high blood pressure’ and ‘severe obesity’—along with a ‘mid risk’ cluster that captured all other participants (Fig. 6 and Supplementary Fig. 1). As the number of clusters increased, more refined and specific groups were identified as subsets of one or more of the existing clusters. For instance, the ‘high cholesterol’ cluster appeared at *k* = 7 for women, with participants coming from the clusters of ‘high blood pressure’ and ‘mid risk’ at *k* = 6. Similarly, the ‘mid risk’ group for men at *k* = 7 split into ‘mid risk tall’ and ‘mid risk short’ at *k* = 8. For both sexes, the ‘severe hyperglycemia’ cluster appeared at *k* = 5 and remained relatively unchanged as *k* increased, as did the ‘low DBP, low eGFR’ cluster after *k* = 6.

## Strengths and limitations

The strengths of our study include using a novel approach to identifying a comprehensive set of epidemiologically and clinically relevant phenotypes that characterizes the entire national population while covering four decades using repeated nationally representative samples with a largely consistent methodology, which allowed measuring change and disparities in phenotype prevalence and its predictors. Our study has some limitations. First, we did not include any inflammation-related biomarkers, such as C-reactive protein, or other cardiometabolic or renal biomarkers, such cystatin C or apolipoprotein B, because these data were not available in some rounds of NHANES. Second, this analysis was based on a series of repeated cross-sectional samples and was not designed to evaluate how an individual with a specific phenotype in one year may have shifted to another in a later year or how the identified phenotypes affect the risk of disease onset or death, which should be pursued with data from prospective cohort studies. Third, other clustering methods should be tested in future methodological assessments, especially probabilistic clustering methods that estimate the probabilities that each participant belongs to each cluster. Finally, although we analyzed some predictors of cluster allocation, future research should investigate how other factors, including genetics, diet, behaviors and the living environment, affect assignment to specific clusters.

## Discussion

Application of data-driven clustering, which has been applied extensively to genomics data, to population-based risk factor data identified a comprehensive set of clinically relevant cardiometabolic and renal phenotypes in the US adult population over a period of four decades. The results showed an increase in the ‘severe obesity’ phenotype whose other cardiometabolic risks were not noticeably different from the average population, a stable prevalence of the ‘severe hyperglycemia’ phenotype and a sharp decrease in the ‘high cholesterol’ and ‘high blood pressure’ phenotypes. This improvement in vascular health has been partly offset by rising prevalence of those with poor kidney function in the ‘low DBP, low eGFR’ cluster.

To our knowledge, no study has applied data-driven clustering methods to repeated nationally representative data to identify multifactorial cardiometabolic and renal phenotypes, and to analyze their trends, in the US population. Our results were consistent with single-risk-factor trend studies on obesity, hypertension or blood lipids, which showed a rise in the former but a decline in the latter two risk factors, including in individuals with obesity^34–36,42,43^. Our result on the higher prevalence of the ‘low risk’ phenotype in women than in men was also consistent with previous findings on cardiovascular health of the US population^44^. We further observed a decrease in the ‘low risk’ phenotype in women and no detectable change for men, which was consistent with a reported statistically insignificant trend in the prevalence of optimal cardiometabolic health for both sexes combined^44^. We did not observe an increase in the ‘severe hyperglycemia’ phenotype between 1988 and 2018 despite the reported rise in diabetes in the United States^71^. This was because the ‘severe hyperglycemia’ phenotype was characterized by very high HbA1c levels and included individuals with uncontrolled diabetes, consistent with previous findings on diabetes subgroups^53,54^. The prevalence of people at such high levels of HbA1c has been relatively stable because improvements in diagnosis and management have countered the rise in total diabetes prevalence^72^. The ‘low DBP, low eGFR’ phenotype, which had two dominant features (high pulse pressure and poor kidney function), is consistent with the association between atherosclerosis and CKD^73^. This phenotype was found predominantly in older ages, had a high prevalence of diabetes and was associated with a history of MI and CHF for women, consistent with high levels of vascular–renal comorbidity in older ages^74^ and with the association of CHF with pulse pressure^75^. The observed increase in the ‘low DBP, low eGFR’ phenotype, especially in the early 2000s, was also consistent with the previously reported rise in the prevalence of CKD in the United States^76^. We did not identify a metabolically healthy obesity phenotype, which accounted for 9.7% of the US population in one study on this specific group^77^, even after allowing 12 clusters to be formed. There may be two reasons for this apparent difference. First, half of the people classified as metabolically healthy in the aforementioned study^77^ had one metabolic risk factor. Second, in our study, such people were clustered either in the ‘severe obesity’ phenotype or in the two mid-risk phenotypes. Finally, our results on ethnic and educational disparities in the prevalence of specific clusters were consistent with previous studies that considered risk factors either individually^36,78^ or through the lens of optimal cardiometabolic health^23^, but these studies did not examine disparities in a comprehensive set of cardiometabolic and renal phenotypes of risk factors. Our results are not directly comparable with those using electronic health records due to differences in the study population, methods and clinical conditions used in the clustering and because some of these studies aimed at identifying subtypes of specific diseases^45,47,48,50,51,53–68^. Among such studies, two studies in different populations identified phenotypes characterized by compromised kidney function and low DBP^50,56^. Another study that used electronic health records in London also found a cluster with both CHF and CKD^62^, which is analogous to our ‘low DBP, low eGFR’ phenotype. One study using electronic health records found a subtype of type 2 diabetes characterized by very high HbA1c levels analogous to the ‘severe hyperglycemia’ phenotype identified in our study^54^.

Our analysis coherently uncovered epidemiological subgroups of the US population characterized by distinct profiles of cardiometabolic and renal risk factors. Some of these phenotypes were characterized by high levels of one or two closely related risk factors, whereas others were more complex and based on multiple seemingly unrelated traits that may share upstream clinical and sociodemographic determinants. Although genetics influences individual or multiple risk factors^79–85^, the risk factors that characterized the clusters identified in our study are also influenced by behavioral, environmental and dietary determinants as well as the use (or non-use) of medicines that lower risk factor levels. Future research combining these determinants with genetic data is needed to discern their contributions to the prevalence and trends in cardiometabolic phenotypes and their influence on the occurrence of disease. Our results apply to the US population, and future research should also compare cardiometabolic and renal phenotypes across populations with different diets, health behaviors, healthcare and genetics.

**Fig. 7:**
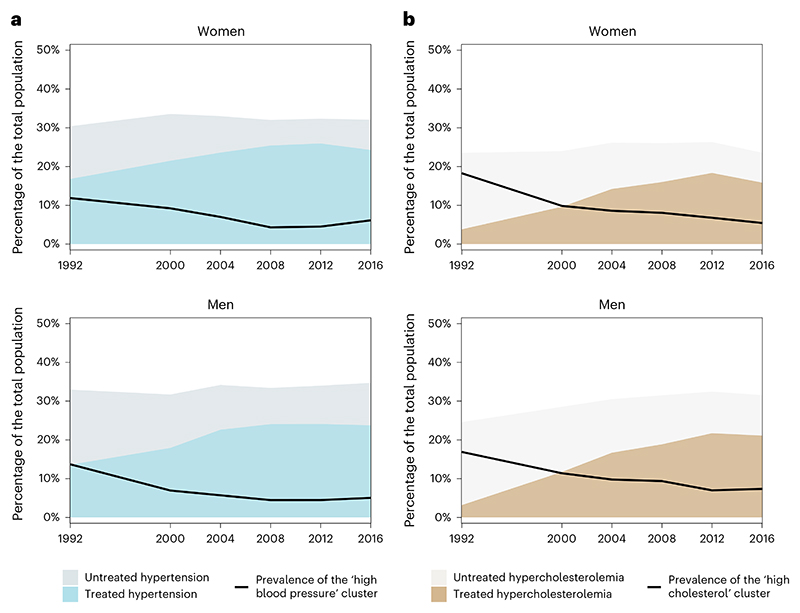
Age-standardized trends in hypertension, treated hypertension and prevalence of the ‘high blood pressure’ phenotype (a) and in hypercholesterolemia, treated hypercholesterolemia and prevalence of the ‘high cholesterol’ phenotype(b). Hypertension is defined as having SBP 140 mmHg or greater, DBP 90 mmHg or greater or taking medication for hypertension. Hypercholesterolemia is defined as having non-HDL 4.92 mmol L^−1^ or greater or taking medication for hypercholesterolemia.

Although the prevalence of the phenotype characterized by very high BMI and WHtR has increased, this group had about average levels of other risk factors. Nonetheless, higher-than-median BMI was also a trait of the ‘severe hyperglycemia’ phenotype, which has not declined despite improvements in diabetes detection and treatment, reflecting the growth of incidence and prevalence of diabetes during the period examined^38^. There was a substantial decline in phenotypes characterized by high levels of non-HDL cholesterol and SBP and DBP, despite the rise in the ‘severe obesity’ phenotype. The use of antihypertensive medicines, which increased over time, may be one of the reasons that those in the ‘severe obesity’ cluster have near-average blood pressure levels despite their high BMI and WHtR levels. The use of statins and antihypertensive medications may have also shifted some treated individuals from the ‘high blood pressure’ and ‘high cholesterol’ groups into the two mid-risk ones, as seen in the correlated trends in the prevalence of the ‘high cholesterol’ and ‘high blood pressure’ phenotypes with the use of statins and antihypertensive medications, respectively (Fig. 7)^86,87^. These improvements have contributed to the decades-long decline in cardiovascular mortality in the United States through lower event rates and better survival^88,89^. The delayed vascular events and better survival, however, may have engendered a rise in an older group with increasingly vascular–renal comorbidities, represented by the ‘low DBP, low eGFR’ phenotype, among whom history of MI and stroke was common and the prevalence of CHF was high. The increase of the ‘high blood pressure’ phenotype since late 2000s may be due to the fact that hypertension treatment and control in the United States, and in other high-income countries, has not improved over the past decade^90^. This stagnation may be partly responsible for the recent deceleration in the decline of CVD mortality^89^. Public health actions, especially those that enhance access to healthier foods, such as fresh fruits and vegetables, legumes and unprocessed grains, as well as treatment of hypertension, high cholesterol and diabetes, can help shift an increasing share of the population from some of the high-risk phenotypes to low-risk and mid-risk ones and delay the onset of comorbid chronic conditions that characterized the ‘low DBP, low eGFR’ phenotype. New medicines for obesity, if their cost is lowered, may also reduce the prevalence of the ‘severe obesity’ phenotype, which has average levels of other risk factors, and also reduce BMI among people who fall in other high-risk clusters^91^. These interventions may be optimized and targeted in the future through precision public health approaches that use the entire risk factor profile or more efficient risk stratification and risk factor management through both clinical and community-based interventions.

## Methods

### Data

The NHANES is a nationally representative survey of the US non-institutionalized civilian population aged 2 months or older with a multistage, stratified clustered probability sample design. The first round of NHANES was done in 1959, and, since 1999, it has been conducted in continuous 2-year rounds. Details of survey design and sampling are provided elsewhere^92^ and are summarized below.

We used 11 rounds of NHANES, including NHANES III (1988–1994) and various rounds of continuous NHANES from 1999 to 2018, for analyzing trends in cardiometabolic and renal traits. We did not use rounds before NHANES III because they did not measure HbA1c. NHANES participants are not re-enrolled in subsequent years, except through chance. Therefore, our results represent cardiometabolic and renal clusters present in successive US populations.

Participants in each round of NHANES were sampled to be collectively representative of the population in the survey year. Ethnic minorities as well as older adults were oversampled to provide stable estimates for these groups. Sample weights were calculated to account for the complex survey design, survey non-response and post-stratification adjustment to match total population counts from the Census Bureau.

We restricted the analysis to participants aged 20 years and older who had all the required biomarker measurements available. We used the following risk factors in our study, based on their relevance to cardiometabolic and renal diseases and their availability in NHANES data.

Anthropometric measures: we used height (cm); BMI, defined as weight divided by height squared (kg m^−2^); and WHtR, defined as waist circumference divided by height. Being taller is associated with a lower risk of CVDs and all-cause mortality but a higher risk of some cancers^13^. High BMI is a risk factor for diabetes, CVDs, several cancers and kidney and liver diseases^9,14^. WHtR was included as a measure of abdominal obesity, which may increase the risk of disease and death independently of BMI^93^.

Blood pressure and heart rate: we used SBP and DBP as they are associated with increased risk of CVDs, kidney disease and dementia^8^. We included resting heart rate (RHR), as higher values have been associated with increased risk of cardiovascular and all-cause mortality^3^. RHR was measured as 60-s pulse and referred to as pulse rate.

Lipids: we used HDL and non-HDL cholesterol defined as total cholesterol (TC) minus HDL cholesterol. Non-HDL cholesterol is associated with higher risk of ischemic heart disease and stroke, and HDL cholesterol is a marker for lower risk^11^.

Glycemia: we used HbA1c as a proxy of average glucose levels in the blood for recent weeks, which has been associated with CVDs^12^, as the marker for glycemic risk and control.

Kidney function: we used eGFR (using the CKD-EPI creatinine equation) as a measure of kidney function, which is a predictor of CKD and CVDs^5,6^.

All the risk factors used in the clustering were measured. Physical examinations were conducted in a mobile examination center, and blood samples were drawn from a random subset of the participants. Blood pressure was measured three times on the right arm with a sphygmomanometer and appropriate cuff size in seated position after a 5-min rest period in all rounds. Both TC and HDL analyses were conducted on venous samples collected according to a standardized protocol. Although there were changes in the laboratories, methods and instruments used to measure lipid concentrations across survey periods were standardized according to the criteria of the Centers for Disease Control and Prevention (CDC) or the National Heart, Lung, and Blood Institute Lipid Standardization Program of the CDC^94^. HbA1c was measured in all NHANES cycles using high-performance liquid chromatography. We followed NHANES recommendations and did not apply any calibration correction based on cross-over regression. Before eGFR calculation, serum creatinine measurements were calibrated using a previously reported calibration equation^95^ to account for potential drift in measurement methods. More information on NHANES measurement, laboratory procedures and careful quality controls can be found on the survey website: http://www.cdc.gov/nchs/nhanes.htm.

We did not use data on inflammation markers, such as C-reactive protein, because these data were only available in some rounds of NHANES. We also used data on age, sex, race and ethnicity, education, history of diseases and medication use for examining the demographic and clinical characteristics of the clusters; these data were collected through a questionnaire.

### Data cleaning

**Extended Data Fig. 4:**
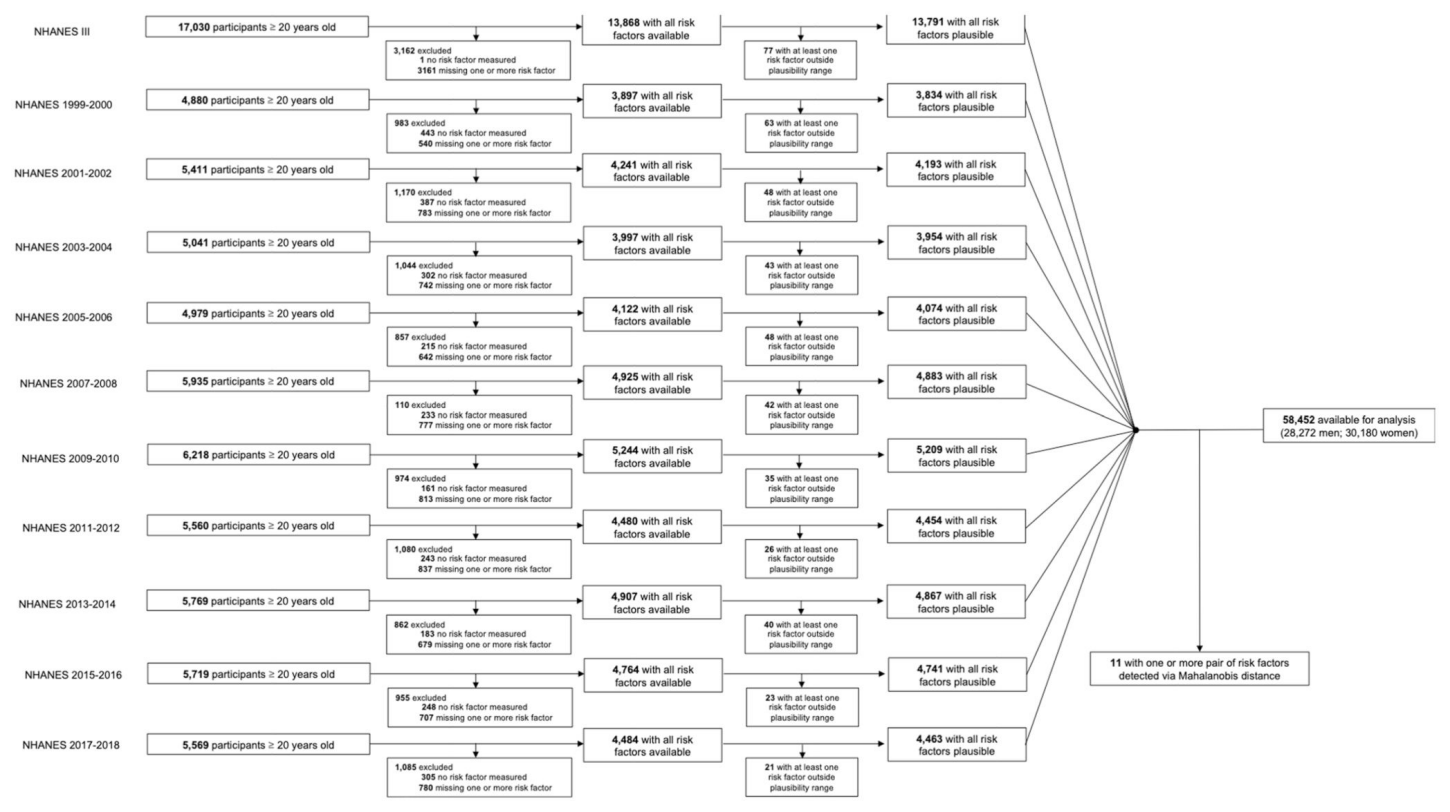
Flowchart of data cleaning. Data cleaning per survey round.

Before analyses, we conducted the following data cleaning procedure. First, we removed measurements outside pre-defined plausibility ranges (Supplementary Table 2). Second, for blood pressure, we discarded the first measurement and used the average of the remaining measurements. Third, for all participants, we confirmed that SBP > DBP and TC ≥ HDL. Finally, we applied an outlier detection procedure based on Mahalanobis distance^96^ to exclude risk factor pairs that had an implausible pairwise relationship relative to the overall data. This method uses the empirical relationship between risk factor pairs to detect extreme combinations, for example, a high SBP of 248 mmHg but low DBP of 40 mmHg or a high BMI of 42 kg m^−2^ but small waist circumference of 74 cm. We applied this technique separately to all pairs of anthropometric variables (height, weight, BMI, waist circumference and WHtR), those of blood pressure (SBP and DBP) and those of lipids (TC and HDL). All variables except height and DBP were log transformed before outlier detection to account for their skewed distributions. For each pair considered, observations with a Mahalanobis distance larger than 40.08 (equivalent to a distance of six standard deviations from the mean) were excluded. The present analysis used data from 58,452 participants (28,272 men and 30,180 women) after applying the above steps (Extended Data Fig. 4).

### Statistical analysis—cluster identification

Our analytical objective was to divide the NHANES sample into groups of participants with risk factor levels that are similar to each other but distinct from those in other clusters. In extreme cases of one or more risk factors—for example, familial hypercholesterolemia or possibly type 1 diabetes—this task is relatively straightforward and may even be feasible based on prior knowledge or visual inspection of data. For national populations, however, such partitioning requires a method that operationalizes the analytical objective by partitioning the joint distribution of risk factors.

We used a *k*-means clustering algorithm to identify cardiometabolic and renal phenotypes of the US population in an unsupervised data-driven approach. The *k*-means algorithm partitions participants into non-overlapping clusters that are relatively homogeneous while maximizing the heterogeneity between clusters, by minimizing the sum of distances of all data points from the center of the cluster they belong to. The *k*-means algorithm is a specific form of Gaussian mixture method where only the means of the clusters are estimated but not their covariance^97^. It is a widely used and computationally efficient clustering algorithm that produces non-overlapping clusters. We took 50 different random sets of starting values to avoid converging to local minima and used Euclidian distance and the Lloyd implementation of the algorithm.

All analyses were conducted by pooling individual participant data across all survey rounds but separately for men and women to allow for potentially different clustering of cardiometabolic traits between them. We centered and scaled each risk factor by subtracting the overall mean and dividing by the standard deviation before clustering. In *k*-means, the number of clusters (*k*) must be pre-specified. Various heuristics have been suggested for selecting the optimal number of clusters—for example, the elbow method and the silhouette method—which compare measures of cluster cohesion and cluster separation for different choices of *k*. Neither the elbow nor the silhouette method provided a definitive optimal number of clusters (Supplementary Fig. 2). Therefore, we investigated cluster membership, and characteristics when sequentially changing *k* from 5 to 12, and selected *k* based on these heuristics as well as on the epidemiological interpretability of the results.

### Stability of the clustering results

After selecting the number of clusters, we evaluated the stability of the resultant clusters by calculating the average Jaccard index^98^ between the clustering results over the entire sample and that of 1,000 sub-samples of 50% of the data drawn without replacement (Extended Data Table 4). The Jaccard index is a measure of similarity between two groups and ranges from 0 to 1, with 0 indicating no overlap and 1 indicating identical results. For men, all clusters had an average Jaccard index of 0.87 or above; for women, all clusters had an average Jaccard index of 0.80 or above, except for the ‘mid risk tall’ phenotype that had an average Jaccard index of 0.70. To evaluate whether our analysis met our analytical objective of partitioning the joint distribution of risk factors based on a true correlation structure, we also used *k*-means to cluster 30,180 simulated data points (the same number as used in the main analysis). The simulated data were generated from a 10-dimensional normal distribution with no correlation. All the resulting clusters were highly unstable with a Jaccard index below 0.30, which is much lower than those of clusters identified on NAHNES data (Extended Data Table 4).

### Intra-cluster and inter-cluster distances

**Extended Data Fig. 5:**
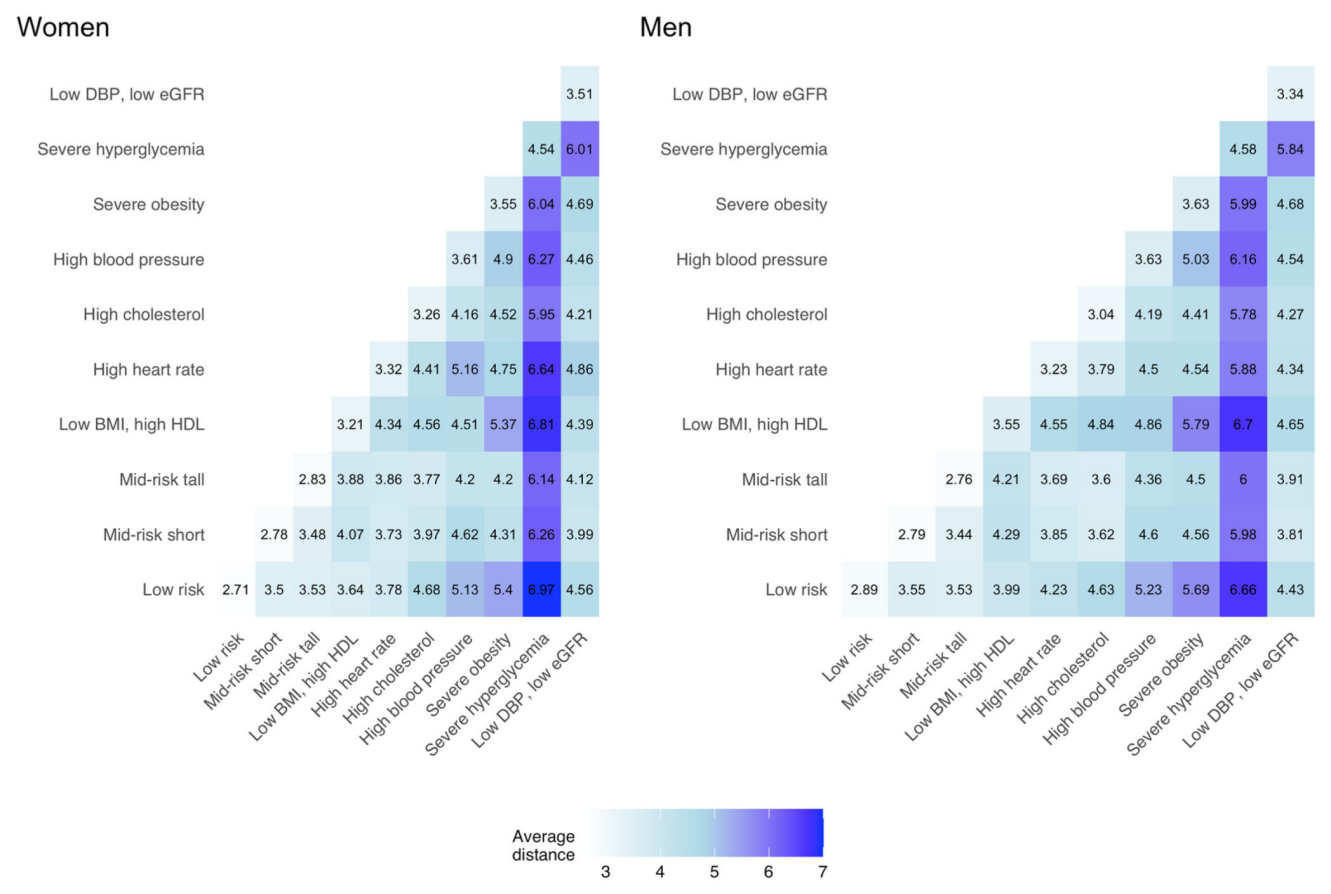
Average intra- and inter-cluster distances for both women and men. Each cell of the diagonal represents the average Euclidian distance between all pairs in a given cluster (Inter-cluster distance). Each cell on the off diagonal represents the average Euclidian distance between all pairs of individuals from different clusters (intra-cluster distance).

We also report (Extended Data Fig. 5) the intra-cluster and inter-cluster distances as a measure of how the method achieves the analytical objective. The intra-cluster distance was calculated as the average Euclidian distance between all pairs of points in the same cluster, and the inter-cluster distance was calculated as the average Euclidian distance between all pairs of points from two different clusters. These metrics show that participants assigned to every cluster were, on average, more similar to one another in terms of their risk factor levels than they were to participants in any other cluster.

### Consistency of clusters over time

We investigated whether clusters emerging from the analysis of all rounds of NHANES from 1988 to 2018 were similar to those that would emerge if we repeated the analysis for subperiods consisting of NHANES III 1988–1994, NHANES 1999–2008 and NHANES 2009–2018 separately (Supplementary Fig. 3). The phenotypes identified in sub-periods were similar to those identified when aggregating all rounds from 1998 to 2018 for men. For women, most of the phenotypes identified over the entire analysis period remained in subperiod clustering, except the ‘mid risk tall’ phenotype, which was replaced by either an ‘obesity’ phenotype or a ‘mid risk’ phenotype, and except the ‘low DBP, low eGFR’ phenotype in NHANES III, which was replaced with a ‘high risk’ phenotype with hazardous levels of all risk factors.

### Statistical analysis—trends in prevalence and predictors of cluster membership

In addition to graphical presentation of how cluster prevalence has changed over time, we analyzed the presence of a trend in a regression analysis. We fitted one logistic regression per cluster, with time as the independent variable. We adjusted for age by 5-year age bands and report the *P* value for the coefficient of the time term. In addition to the entire analysis period, we analyzed trends for pre-specified time periods of 1988–2000, 2000–2010 and 2010–2018 (Extended Data Table 3).

We also used multivariate logistic regression to analyze the predictors of cluster membership. The predictors included age group, survey year, race or ethnicity (non-Hispanic White, non-Hispanic Black, Hispanic and Other ethnicity), education (below high school, high school and university or college), medication use (antihypertensive, statin, oral hypoglycemic diabetes medication and insulin), smoking (current smoking, never smoking and former smoking) and previous history of disease (MI, stroke and CHF).

When reporting the prevalence of clusters over time, and the potential predictors of cluster membership, we accounted for the sampling design through the use of sample weights in the regressions. In all regressions, we rescaled sample weights so that they summed to the same total in each round. We did this so that each round of NHANES contributes the same effective sample size to the analysis of trends and predictors. When evaluating trends over time and predictors of cluster membership, we also adjusted the sample weights by 5-year age bands to match the age distribution of the 2020 US census population. All analysis were done using R software version 4.0.3

### Reporting summary

Further information on research design is available in the Nature Portfolio Reporting Summary linked to this article.

## Extended Data

**Extended Data Table 1:** Clinical characteristics of cardiometabolic and renal clusters

*Women*	Hypertension^a^	Diabetes^b^		Obesity^c^		History of Myocardial Infarction	History of Stroke	History of Congestive Heart Failure
Low risk n=4,632	2.5% (2.1-3.0) n=117	0.7% (0.5-1.0) n=33		0.7% (0.5-1.0) n=33		0.6% (0.5-0.9) n=30	0.8% (0.5-1.1) n=35	0.3% (0.2-0.6) n=16
Mid-risk short n=3,942	8.2% (7.3-9.1) n=321	4.9% (4.3-5.6) n=192		36.8% (35.3-38.3) n=1,449		0.9% (0.6-1.2) n=34	1.1% (0.8-1.4) n=41	0.7% (0.5-1.1) n=29
Mid-risk tall n=3,715	25.2% (23.8-26.6) n=933	5.6% (4.9-6.4) n=207		44.1% (42.5-45.7) n=1,638		1.3% (1.0-1.7) n=47	2.2% (1.7-2.7) n=80	1.1% (0.8-1.5) n=41
Low BMI, high HDL n=3,027	32.9% (31.2-34.6) n=994	3.8% (3.2-4.6) n=116		6.6% (5.8-7.6) n=201		2.0% (1.5-2.5) n=59	2.4% (2.0-3.1) n=74	1.3% (1.0-1.8) n=40
High heart rate n=2,591	7.2% (6.3-8.3) n=187	3.2 (2.6-3.9) n=82		29.5% (27.8-31.3) n=765		0.7% (0.5-1.2) n=19	1.0% (0.7-1.4) n=25	0.4% (0.2-0.7) n=10
High cholesterol n=2,751	42.5% (40.7-44.4) n=1,167	12.5% (11.3-13.8) n=343		40.7% (38.9-42.6) n=1,120		3.8% (3.1-4.5) n=103	4.0% (3.3-4.8) n=110	3.0% (2.4-3.7) n=82
High blood pressure n=2,522	95.6% (94.7-96.3) n=2,410	14.8% (13.4-16.2) n=372		36.2% (34.4-38.1) n=913		4.2% (3.5-5.0) n=105	6.2% (5.4-7.3) n=157	3.7% (3.0-4.5) n=93
Severe obesity n=3,247	44.1% (42.4-45.8) n=1,428	22.2% (20.8-23.6) n=720		99.9% (99.8-100.0) n=3,245		2.2% (1.7-2.7) n=70	3.0% (2.4-3.6) n=96	3.4% (2.8-4.0) n=109
Severe hyperglycemia n=886	58.1% (54.8-61.3) n=514	100.0% (--)^d ^n=886		64.1% (60.9-67.2) n=568		6.9% (5.4-8.8) n=61	5.6% (4.3-7.4) n=50	6.4% (5.0-8.2) n=57
Low DBP, low eGFR n=2,867	75.6% (74.0-77.1) n=2,163	31.2% (29.5-32.9) n=894		43.3% (41.5-45.1) n=1,241		8.9% (7.9-10.0) n=253	8.4% (7.5-9.5) n=241	9.0% (8.0-10.1) n=257
All n=30,180	34.0% (33.4-34.5) n=10,234	12.8% (12.4-13.1) n=3,845		37.0% (36.5-37.6) n=11,173		2.6% (2.4-2.8) n=781	3.0% (2.8-3.2) n=909	2.4% (2.3-2.6) n=734
*Men*	Hypertension^a^		Diabetes^b^		Obesity^c^	History of Myocardial Infarction	History of Stroke	History of Congestive Heart Failure
Low risk n=3,730	4.3% (3.7-5.0) n=159		1.5% (1.2-1.9) n=56		0.% (0.4-0.9) n=21	1.4% (1.1-1.8) n=51	0.7% (0.5-1.0) n=25	0.7% (0.5-1.1) n=27
Mid-risk short n=3,981	9.0% (8.2-10.0) n=359		4.9% (4.3-5.6) n=194		20.1% (18.9-21.4) n=801	2.0% (1.7-2.5) n=81	1.2% (0.9-1.6) n=48	1.0% (0.7-1.4) n=40
Mid-risk tall n=4,028	21.2% (20.0.9-22.5) n=852		4.0% (3.5-4.7) n=163		23.0% (21.7-24.3) n=925	2.7% *(22-3.2) *n=107	1.4% (1.1-1.8) n=57	1.6% (1.3-2.1) n=65
LowBMI, high HDL n=2,174	38.8% (36.8-40.9) n=842		5.7% (4.8-6.8) n=124		4.9% (4.1-5.9) n=106	4.4% (3.6-5.3) n=95	2.8% (2.2-3.5) n=60	2.4% (1.8-3.1) n=52
High heart rate n=2,401	33.5% (31.6-35.4) n=803		10.4% (9.2-11.6) n=248		27.2% (25.4-29.0) n=652	3.9% (3.2-4.8) n=94	2.5% (2.0-3.3) n=61	2.6% (2.0-3.3) n=62
High cholesterol n=2,887	34.9% (33.2-36.7) n=1,008		8.2% (7.3-9.3) n=237		38.9% (37.1-40.7) n=1,123	4.7% (4.0-5.5) n=135	1.7% (1.3-2.2) n=49	2.2% (1.7-2.8) n=64
High blood pressure n=2,396	97.9% (97.3-98.4) n=2,346		17.7% (16.2-19.3) n=423		26.4% (24.7-28.2) =632	10.5% (9.3-11.8) n=250	7.4% (6.5-8.6) n=178	6.5% (5.6-7.5) n=154
Severe obesity n=2,613	51.7% (49.8-53.7) n=1,350		25.1% (23.5-26.8) n=655		100.0% (--)^d ^n=2,613	6.4% (5.5-7.4) n=166	3.1% (2.5-3.9) n=82	4.9% (4.2-5.9) n=129
Severe hyperglycemia n=976	53.7% (50.6-56.9) n=524		100.0% (--)^d ^n=976		47.6% (44.5-50.8) n=465	10.6% (8.8-12.7) n=103	5.9% (4.6-7.5) n=57	6.8% (5.4-8.6) n=66
Low DBP, low eGFR n=3,086	59.5% (57.8-61.2) n=1,830		31.2% (29.6-32.9) n=964		34.6% (33.0-36.3) n=1,068	18.7% (17.4-20.1) n=575	9.3% (8.3-10.4) n=286	11.5% (10.5-12.7) n=353
All n=28,272	35.7% (35.1-36.3) n=10,073		14.3% (13.9-14.7) n=4,040		29.7% (29.2-30.3) n=8,406	5.9% (5.6-6.2) n=1,657	3.2% (3.0-3.4) n=903	3.6% (3.4-3.8) n=1,012

a Obesity is defined as BM >30 kg/m^2^

b Hypertension is defined as SBP 2140 mmHg. DBP *90 mmHg or taking antihypertensive drug

c Diabetes is defined as either HbA1c 26.5%, or use of insulin or oral hypoglycemic drugs.

d Confidence interval are not applicable where the corresponding where the corresponding number of individuals is less than The numbers show prevalence in each cluster. Numbers in parentheses indicate the 95% confidence interval, and numbers on the second row represent the corresponding number of individuals. Confidence intervals were calculated using the Wilson score method.

**Extended Data Table 2:** Medication use in cardiometabolic and renal clusters of US adults for 2011–2018 NHANES rounds

*Women*	Medication use			
	Antihypertensive	Statins	Oral hypoglycemic	Insulin
Low risk n=1,347	3.5% (2.7-4.6) n=47	3.8% (2.9-5.0) n=51	0.4% (--)^a ^n=5	0.0% (--)^a ^ n=0
Mid-risk short n=1,400	11.3% (9.7-13.1) n=150	8.0% (6.7-9.6) n=107	3.9% (3.0-5.1) n=52	0.3% (--)^a ^ n=4
Mid-risk tall n=1,129	25.0% (22.6-27.7) n=277	14.8% (12.8-17.0) n=164	4.1% (3.1-5.5) n=46	0.5% (--)* n=5
Low BMI, high HDL n=997	24.6% (22.0-27.4) n=239	16.5% (14.3-19.0) n=160	2.6% (1.8-3.8) n=25	0.3% (--)^a ^ n=3
High heart rate n=639	9.6% (7.6-12.0) n=65	5.3% (3.9-7.3) n=36	2.9% (1.9-4.5) n=20	0.7% (--)^a ^ n=5
High cholesterol n=574	31.1% (27.4-35.0) n=174	17.9% (14.9-21.3) n=100	7.3% (5.4-9.8) n=41	1.4% (--)^a ^ n=8
High blood pressure n=621	52.7% (48.3-57.1) n=263	28.2% (24.4-32.3) n=141	8.8% (6.6-11.6) n=44	3.0% (1.8-4.9) n=15
Severe obesity n=1,385	38.9% (36.2-41.6) n=490	17.9% (15.9-20.1) n=226	13.6% (11.8-15.6) n=171	3.7% (2.8-4.8) n=47
Severe hyperglycemia n=301	45.0% (39.2-50.8) n=125	38.1% (32.6-44.0) n=106	54.7% (48.8-60.5) n=151	45.0% (39.2-50.8) n=125
Low DBP, low eGFR n=1,058	66.9% (64.0-69.7) n=694	50.3% (47.2-53.3) n=519	19.8% (17.5-22.3) n=205	7.9% (6.4-9.7) n=82
All 11=9,451	27.8% (26.9-28.8) n=2,524	17.8% (17.0-18.6) n=1,610	8.4% (7.8-9.0) n=760	3.2% (2.9-3.6) n=294
*Men*	Medication use			
	Antihypertensive	Statins	Antihypertensive	Insulin
Low risk n=1,246	4.6% (3.6-5.9) n=57	4.3% (3.3-5.6) n=54	1.1% (0.7-1.9) n=14	0.6% (--)^a ^ n=7
Mid-risk short n=1,327	9.3% (7.9-11.0) n=123	9.9% (8.4-11.6) n=131	3.8% (2.9-4.9) n=50	0.7% (--)^a ^ n=9
Mid-risk tall n=1,125	17.5% (15.4-19.9) n=197	14.1% (12.2-16.3) n=159	3.1% (2.3-4.3) n=35	1.1% (0.6-1.9) n=12
Low BMI, high HDL n=749	25.7% (22.7-28.9) n=192	18.7% (16.1-21.7) n=140	4.4% (3.2-6.1) n=33	0.9% (--)^a ^ n=7
High heart rate n=736	21.5% (18.7-24.6) n=158	16.7% (14.2-19.6) n=123	7.6% (5.9-9.8) n=56	1.9% (1.1-3.2) n=14
High cholesterol n=638	18.8% (16.0-22.1) n=120	8.5% (6.5-10.9) n=54	3.6% (2.4-5.4) n=23	0.3% (--)^a ^ n=2
High blood pressure n=586	45.6% (41.6-49.6) n=267	27.1% (23.7-30.9) n=158	13.8 (11.3-16.9) n=81	3.8% (2.5-5.6) n=22
Severe obesity n=1,128	37.6% (34.8-40.5) n=424	25.2% (22.7-27.8) n=283	16.4% (14.4-18.7) n=185	5.1%(4.0-6.6) n=58
Severe hyperglycemia n=347	43.2% (38.1-48.5) n=149	43.0% (37.8-48.3) n=147	59.5% (54.3-64.6) n=206	38.0% (33.1-43.3) n=132
Low DBP, low eGFR n=1,192	57.9% (55.1-60.7) n=689	55.7% (52.8-58.5) n=659	26.4% (24.0-29.0) n=315	9.0% (7.5-10.7) n=107
All n=9,074	26.2% (25.3-27.2) n=2,376	21.1% (20.3-21.9) n=1,908	11.0% (10.4-11.7) n=998	4.1% (3.7-4.5) n=370

a Confidence interval are not applicable where the corresponding number of individuals is less than 10. Each cell shows percent of cluster participants that use a specific medication. Numbers in parentheses indicate the 95% confidence interval, and numbers on the second row represent the corresponding number of individuals. Confidence intervals were calculated using the Wilson score method.

**Extended Data Table 3:** Changes in phenotypes prevalence in pre-specified periods

*Women*	1988-2000			2000-2010			2010-2018		
	Percentage change in odds per year		P values	Percentage change in odds per year		P values	Percentage change in odds per year		P values
Low risk	-1		5.07e-01	-0.6		6.05E-01	-1.9		1.83e-01
Mid-risk short	13.1		4.83e-09	-1.3		2.73E-01	0.6		6.44e-01
Mid-risk tall	-2.1		9.33e-02	0.4		6.49E-01	-1.6		2.69e-01
LowBMI, high HDL	-5.1		5.00e-03	3		1.13E-02	0.6		6.79e-01
High heart rate	9.9		3.20e-06	0.4		7.61E-01	-4.9		9.09e-03
High cholesterol	-7.8		5.19e-05	-2.8		5.11E-02	-5.8		4.03e-03
High blood pressure	-4.1		4.86e-03	-9.5		1.42E-10	8.4		1.49e-03
Severe obesity	7.8		8.64e-05	3.3		9.77E-03	5.3		8.77e-04
Severe hyperglycemia	2.3		2.90e-01	-7.6		5.35E-04	1.5		5.43e-01
Low DBP, low eGFR	1.2		3.84e-01	8.7		3.15E-07	0.1		9.64Ee-01
*Men*	1988-2000			2000-2010			2010-2018		
	Percentage change in odds per year	P values		Percentage change in odds per year	P values		Percentage change in odds per year	P values	
Low risk	3.2	4.28e-02		-1.1	3.29e-01		-0.8	5.80e-01	
Mid-risk short	11.4	6.04e-12		-2	9.77e-02		-0.4	8.24e-01	
Mid-risk tall	1.3	2.24e-01		-1.4	1.56e-01		-4	1.19e-02	
LowBMI, high HDL	-2.1	1.57e-01		2.3	8.63e-02		0.5	8.12e-01	
High heart rate	-6.6	2.60e-04		2.5	4.10e-02		-1.4	3.61e-01	
High cholesterol	-5.6	2.84e-06		-3	9.54e-03		-1.7	4.35e-01	
High blood pressure	-8.9	5.53e-08		-6	2.75e-06		5	3.46e-02	
Severe obesity	10.9	1.02e-06		3.1	1.10e-02		5.6	1.26e-03	
Severe hyperglycemia	2.3	2.73e-01		-3.1	1.42e-01		2.6	3.28e-01	
Low DBP, low eGFR	-3.9	3.30e-02		9.7	4.39e-10		-0.1	9.72e-01	

We accounted for the sampling design through the use of sample weights. For each round, sample weights were adjusted by 5-year age bands to add to the same number. *P* values were obtained from a two-sided *t*-test and were not adjusted for multiple comparison.

**Extended Data Table 4:** Average Jaccard index of the clusters identified using NHANES data and on those identified using simulated data from independent normal distribution

Women	
Cluster names	Average Jaccard index
Low risk	0.80
Mid-risk short	0.80
Mid-risk tall	0.70
Low BMI, high HDL	0.88
High heart rate	0.80
High cholesterol	0.83
High blood pressure	0.88
Severe obesity	0.89
Severe hyperglycemia	0.97
Low DBP, low eGFR	0.91

Men	
Cluster names	Average Jaccard index
Low risk	0.93
Mid-risk short	0.90
Mid-risk tall	0.90
Low BMI, high HDL	0.93
High heart rate	0.87
High cholesterol	0.88
High blood pressure	0.94
Severe obesity	0.95
Severe hyperglycemia	0.97
Low DBP, low eGFR	0.93

Simulated Data	
Cluster	Average Jaccard index
Cluster 1	0.21
Cluster 2	0.25
Cluster 3	0.27
Cluster 4	0.25
Cluster 5	0.23
Cluster 6	0.29
Cluster 7	0.22
Cluster 8	0.22
Cluster 9	0.23
Cluster 10	0.27

The Jaccard index was calculated over 1,000 subsamples without replacement of 50% of the data.

## Supplementary Material

Supplementary Tables

Reporting Summary

## Figures and Tables

**Table 1:** Demographic characteristics and medication use of cardiometabolic and renal clusters of US adults

Women	Age distribution					Medication use^a^			
	Median (years)	20-39years	40-59years	60+years		Antihypertensive	Statins	Oral hypoglycemic	Insulin
Low risk *n* = 4,632	31 (25–40)	73% *n* = 3,404	22% *n* = 1,031	4%*n* = 197		2.3% (1.9–2.8) *n* = 107	2.4% (2.0–2.8) *n* = 109	0.3% (0.2–0.5) *n* = 12	0.1%(–)^a^ *n* = 6
Mid risk short *n* = 3,942	36 (28–46)	59% *n* = 2,333	32% *n* = 1,269	9%*n* = 340		7.6% (6.8–8.4) *n* = 298	5.8% (5.1–6.5) *n* = 228	3.0% (2.5–3.6) *n* = 119	0.4% (0.2–0.6) *n* = 14
Mid risk tall *n* = 3,715	45 (36–55)	35%*n* = 1,309	46% *n* = 1711	19%*n* = 695		20.1% (18.9–21.4)*n* = 745	10.0% (9.1–11.1) *n* = 373	2.7% (2.3–3.3) *n* = 101	0.7% (0.5–1.0) *n* = 26
Low BMI, high HDL *n* = 3,027	55 (45–66)	19%*n* = 578	40% *n* = 1,202	41% *n* = 1,247		21.4% (19.9.0–22.9) *n* = 646	11.7% (10.7–13.0) *n* = 355	1.9% (1.4–2.4) *n* = 56	0.3% (0.2–0.6) *n* = 10
High heart rate *n* = 2,591	30 (24–38)	77% *n* = 1,993	17%*n* = 452	6%*n* = 146		5.8% (5.0–6.8) *n* = 150	3.0% (2.4–3.7) *n* = 77	1.6% (1.1–2.1) *n* = 41	0.7% (0.5–1.1) *n* = 19
High cholesterol *n* = 2,751	59 (48–68)	12%*n* = 337	39% *n* = 1,069	49% *n* = 1,345		28.7% (27.0–30.4) *n* = 787	13.0% (11.8–14.3) *n* = 358	5.3% (4.5–6.2)*n* = 145	1.5% (1.1–2.0) *n* = 41
High blood pressure *n* = 2,522	64 (54–74)	5%*n* = 121	31%*n* = 774	65% *n* = 1,627		47.1% (45.1–49.0) *n* = 1,184	16.6% (15.2–18.1) *n* = 418	7.0% (6.1–8.1)*n* = 177	2.3% (1.8–3.0) *n* = 58
Severe obesity *n* = 3,247	45 (34–58)	38% *n* = 1,231	40% *n* = 1,293	22% *n* = 723		35.0% (33.4–36.7) *n* = 1,134	14.5% (13.4–15.8)*n* = 471	12.3% (11.2–13.4)*n* = 398	3.5% (2.9–4.1) *n* = 112
Severe hyperglycemia *n* = 886	58 (48–66)	10% *n* = 92	43%*n* = 379	47% *n* = 415		44.1% (40.9–47.4) *n* = 389	25.8% (23.0–28.7) *n* = 228	54.4% (51.1–57.7) *n* = 480	36.3%(33.2–39.6)*n* = 322
Low DBP, low eGFR *n* = 2,867	74 (67–80)	1%*n* = 15	7% *n* = 212	92% *n* = 2,640		60.7% (58.9–62.5) *n* = 1,733	38.0% (36.2–39.8) *n* = 1,085	18.5% (17.1–20.0) *n* = 530	7.5% (6.6–8.5) *n* = 215
All*n* = 30,180	46 (32–63)	38% *n* = 11,413	31% *n* = 9,392	31% *n* = 9,375		23.8% (23.3–24.3) *n* = 7,173	12.3% (11.9–12.7) *n* = 3,702	6.8% (6.6–7.1) *n* = 2,059	2.7% (2.6–2.9)*n* = 823
**Men**		**Age distribution**					**Medication use^a^**		
	**Median (years)**	**20–39years**	**40–59years**	**60+years**		**Antihypertensive**	**Statins**	**Oral hypoglycemic**	**Insulin**
Low risk *n* = 3,730	28 (23–38)	77% *n* = 2,875	16%*n* = 615	6% *n* = 240		2.7% (2.1–3.1) *n* = 100	2.5% (2.2–3.2) *n* = 95	0.7% (0.5–11) *n* = 27	0.5% (0.4–0.8)*n* = 20
Mid risk short *n* = 3,981	38 (28–50)	54% *n* = 2,133	33% *n* = 1,313	13% *n* = 535		6.9% (6.2–7.8) *n* = 274	6.8% (6.1–7.7)*n* = 272	2.6% (2.1–3.1) *n* = 102	0.5% (0.3–0.7) *n* = 18
Mid risk tall *n* = 4,028	44 (33–56)	39% *n* = 1,586	41% *n* = 1,650	20% *n* = 792		14.2% (13.2–15.3) *n* = 572	11.0% (10.1–12.0) *n* = 442	2.0% (1.6–2.5) *n* = 81	0.6% (0.4–0.9) *n* = 26
Low BMI, high HDL *n* = 2,174	54 (39–67)	25% *n* = 544	34%*n* = 747	41% *n* = 883		19.9% (18.3–21.6)*n* = 431	11.7% (10.4–13.1) *n* = 254	3.2% (2.5–4.0) *n* = 69	1.1% (0.7–1.6) *n* = 23
High heart rate *n* = 2,401	45 (34–60)	37% *n* = 889	37% *n* = 899	26% *n* = 613		17.9% (16.4–19.5) *n* = 428	11.6% (10.4–13.0) *n* = 279	6.0% (5.1–7.0) *n* = 144	1.6% (1.2–2.2) *n* = 38
High cholesterol *n* = 2,887	48 (39–60)	26% *n* = 755	48% *n* = 1,378	26% *n* = 754		15.8% (14.5–17.2) *n* = 455	8.2% (7.2–9.3)*n* = 236	2.8% (2.3–3.5) *n* = 82	0.4% (0.2–0.7) *n* = 12
High blood pressure *n* = 2,396	66 (56–74)	5%*n* = 112	25% *n* = 602	70% *n* = 1,682		43.1% (41.2–45.1)*n* = 1,030	16.0% (14.6–17.6) *n* = 383	7.5% (6.5–8.7) *n* = 180	2.9% (2.2–3.7)*n* = 70
Severe obesity *n* = 2,613	48 (35–60)	33%*n* = 868	39% *n* = 1,020	28% *n* = 725		36.5% (34.6–38.3) *n* = 951	20.3% (18.8–21.9) *n* = 530	14.2% (12.9–15.6)*n* = 370	4.4% (3.7–5.2) *n* = 114
Severe hyperglycemia *n* = 976	57 (48–65)	11% *n* = 105	42% *n* = 413	47%*n* = 458		36.8% (33.8–39.9)*n* = 358	31.8% (29.0–34.8)*n* = 309	54.8% (51.6–57.9)*n* = 533	30.6% (27.8–33.5) *n* = 298
Low DBP, low eGFR *n* = 3,086	73 (66–80)	2%*n* = 63	8%*n* = 248	90% *n* = 2,775		51.1% (49.3–52.9) *n* = 1,571	40.5% (38.8–42.3) *n* = 1,244	20.2% (18.8–21.7) *n* = 623	6.7% (5.9–7.7)*n* = 207
All *n* = 28,272	48 (34–64)	35% *n* = 9,930	31% *n* = 8,885	34% *n* = 9,457		21.9% (21.4–22.4)*n* = 6,171	14.3% (13.9–14.7) *n* = 4,044	7.8% (7.5–8.2) *n* = 2,211	2.9% (2.7–3.1) *n* = 826

Each cell shows the percent of cluster participants in all rounds of NHANES used in the analysis that fall in this category. Percent of participants in each cluster allocated to each of the three age groups sum to 100% for each cluster. Numbers in parentheses indicate interquartile range for median age and 95% confidence interval for medication use. Confidence intervals were calculated using the Wilson score method^99^. See Extended Data Table 2 for medication use for the post-2010 period.

a Confidence intervals are not applicable where the corresponding number of individuals is less than 10.

## Data Availability

The data used for this analysis are publicly available and can be downloaded on the NHANES website: https://wwwn.cdc.gov/nchs/nhanes/Default.aspx.

## Code availability

The computer code for the clustering and the multivariate analysis in this work is available at https://globalenvhealth.org/code-data-download/ and https://doi.org/10.5281/zenodo.10075387.
