# Author Correction: Cardiometabolic and renal phenotypes and transitions in the United States population

**DOI:** 10.1038/s44161-024-00425-z

**Published:** 2024-01-15

**Authors:** Victor P. F. Lhoste, Bin Zhou, Anu Mishra, James E. Bennett, Sarah Filippi, Perviz Asaria, Edward W. Gregg, Goodarz Danaei, Majid Ezzati

**Affiliations:** 1 Department of Epidemiology and Biostatistics, School of Public Health, Imperial College London, London, UK; 2 MRC Centre for Environment and Health, School of Public Health, Imperial College London, London, UK; 3 Abdul Latif Jameel Institute for Disease and Emergency Analytics, Imperial College London, London, UK; 4 Department of Mathematics, Imperial College London, London, UK; 5 School of Population Health, Royal College of Surgeons in Ireland, Dublin, Ireland; 6 Department of Global Health and Population, Harvard T.H. Chan School of Public Health, Boston, MA USA; 7 Department of Epidemiology, Harvard T.H. Chan School of Public Health, Boston, MA USA; 8 Regional Institute for Population Studies, University of Ghana, Accra, Ghana

**Keywords:** Cardiology, Machine learning

Correction to: *Nature Cardiovascular Research* 10.1038/s44161-023-00391-y, published online 15 December 2023.

In the version of this article initially published, incorrect versions of Extended Data Figs. 1 and 2, with mismatched data and labels, were presented. The figures have been corrected in the HTML and PDF versions of the article.

